# Combining ability analysis and heterosis for antioxidants, carotenoids and shelf life in papaya (*Carica papaya* L.)

**DOI:** 10.3389/fpls.2026.1920679

**Published:** 2026-10-26

**Authors:** Preeti Singh, Jai Prakash, Sanjay Kumar Singh, Amit Kumar Goswami, Kanhaiya Singh, Satyabrata Pradhan, Chaithra T. S., Laya Prasanna, Zakir Hussain, Naveen Kumar Maurya, Hidayatullah Mir, Subodh Kumar Sinha

**Affiliations:** 1 Department of Horticulture (Fruit Science), Bihar Agricultural University, Sabour, India; 2 Division of Fruits and Horticultural Technology, Indian Agricultural Research Institute (ICAR), New Delhi, India; 3 Indian Council of Agricultural Research, New Delhi, India; 4 Division of Vegetable Science, Indian Agricultural Research Institute (ICAR), New Delhi, India; 5 Department of Fruit Science, College of Horticulture and Forestry, Acharya Narendra Deva University of Agriculture and Technology, Kumarganj, India; 6 Regional Horticulture Research Sub-Station, Bhaderwah, Sher-e-Kashmir University of Agricultural Sciences and Technology of Jammu, Jammu and Kashmir, India; 7 ICAR-National Institute for Plant Biotechnology, New Delhi, India

**Keywords:** diallel analysis, general combining ability, heterosis, nutraceutical traits, papaya, specific combining ability, storability

## Abstract

Papaya, a prominent tropical fruit crop in India, is witnessing expanding cultivation across the country’s diverse agro-climatic zones. This growing popularity is due to its nutritional value, year-round production and economic significance. However, the availability of varieties with improved nutritional and fruit quality remains limited. The trial to investigate combining ability and heterosis for nutritional and eating-quality traits in papaya was conducted at the ICAR-Indian Agricultural Research Institute, New Delhi. The quality traits under study were total soluble solids (TSS), total phenolic content, total flavonoid content, antioxidant activity, β-carotene, lycopene, total carotenoids, fruit firmness, and shelf life. 6 inbred lines of papaya were crossed in a full diallel mating design to develop 30 F1 hybrids, including the reciprocal crosses. The per se mean performance and general combining ability led to the identification of the best parental lines, like P-9–12 for TSS, total carotenoids, β-carotene, lycopene, antioxidants, fruit firmness, and shelf-life, and P-9–5 for total phenolic and flavonoid content. The range of heterosis was low to moderate in papaya hybrids for the traits studied, except for total carotenoids and lycopene, where a high range of heterosis was recorded. The best performing hybrids and specific combiners like PN x P-7–2 for TSS, PS3 x P-7–9 and P-9–5 x PS3 for total phenolic content, P-9–12 x P-9–5 and P-7–2 x P-7–9 for total flavonoid content, PS3 x P-7–2 for antioxidant, P-7–2 x P-9–5 for lycopene, P-7–2 x PN for β-carotene, PS3 x P-7–9 for total carotenoids and P-7–2 x PS3 for fruit firmness and shelf life were identified. Hybrids exhibiting high SCA effects along with desirable mean performance may serve as promising candidates for future hybrid development programs. The study indicated the importance of both additive and non-additive gene action in governing quality and nutritional traits. It demonstrated substantial scope for developing nutritionally superior papaya hybrids with improved shelf life and consumer acceptability.

## 1 Introduction

Papaya (*Carica papaya* L.) is one of the most commercially important fruit cropsin the family Caricaceae and the only species in the genus *Carica*. It is native to South America but can be grown successfully in tropical and subtropical regions worldwide. The crop’s high production potential and nutrient-dense fruits have encouraged papaya cultivation worldwide. India has emerged as the world’s leading producer of papaya, accounting for 39% of global papaya production (FAOSTATFAO, 2025), with an area of 148.92 thousand hectares and a production of 5292.17 thousand tons (Department of Agriculture & Farmers Welfare, 2026). The papaya plant is a short-lived perennial herb of the tropics known for its high yield potential, year-round fruit production, delicious, nutritious fruit, and therapeutic properties. The fruits are a rich source of vitamins A, B, and C, minerals such as phosphorus, magnesium, potassium, and calcium, and fiber (Wall, 2006). In developing countries, papayas are an important source of Vitamin A, which is consumed to address Vitamin A deficiency (Saeed et al., 2014). The fruit is also rich in carotenoids, phenols, flavonoids, and other antioxidants, which are known to confer nutraceutical properties and reduce the risk of many chronic diseases, such as heart disease, cancer, diabetes, and rheumatoid arthritis (Wall, 2006). Additionally, it supports immune health, has anti-inflammatory properties, promotes lung health, aids in blood platelet formation, and is valued as a beauty-enhancing fruit. Carotenoids, which contribute yellow, orange, and red pigments, are abundant in many yellow-orange and red-colored fruits and vegetables such as pumpkin, papaya, mango, tomato, muskmelon, and watermelon. In papaya, the major carotenoid pigments found are β-carotene, β-cryptoxanthin, and lycopene (Wall and Tripathi, 2014). Lycopene is primarily found in red or pink pulp varieties, and β-carotene and β-cryptoxanthin are primarily found in yellow pulp varieties. The factor that influences consumer acceptability of papaya fruit is its eating quality, which is an amalgamation of factors like flavor, aroma, and texture. Luz et al. (2018), while assessing the sensory and quality traits of papaya hybrid fruits, found that texture had a strong positive correlation with aroma and flavor and was identified as an important trait influencing consumer preference. Papaya, a climacteric fruit with thin skin, is highly perishable. Thus, increasing the postharvest shelf life of papaya is another important trait to target. The postharvest shelf life of fruits is directly correlated to fruit firmness, as enhanced firmness maintains cell integrity and ultimately enhances fruit shelf life. To date, most research has focused on enhancing papaya yield and resistance to biotic and abiotic stresses, with little attention to improving fruit quality. However, in recent years, nutritional and eating-quality traits have gained considerable importance in papaya breeding programs. The combined fruit quality and nutritional traits of papaya are closely related to the genotype, environment, and cultivation practices. However, genotype plays the most significant role in determining the nutritional and nutraceutical quality of fruits. Improvement in these traits can be achieved through the selection and development of superior genotypes with higher concentrations of bioactive compounds, enhanced antioxidant potential, and improved fruit quality traits.

Breeding papaya varieties with enhanced fruit and nutritional quality poses significant challenges for breeders because a balance among high yield, superior fruit quality, and disease resistance must be maintained simultaneously. Papaya is among the few fruit crops that are commercially propagated by seed and complete their life cycle in a short period of time. This enables exploitation of heterosis, in which the F1 hybrid is superior to the parental lines. Development of superior F_1_ hybrids with enhanced levels of TSS, vitamins, antioxidants, firmness, and extended shelf life requires a thorough understanding of genetic general combining ability (GCA), specific combining ability (SCA), and the extent of heterosis governing these traits, together with multivariate approaches such as principal component analysis (PCA) to elucidate the overall relationships among quality traits and identify promising parental and hybrid combinations based on their multidimensional performance. The GCA would reveal suitable parents for the traits of interest, whereas the SCA and heterosis would aid in identifying the best-performing hybrids. In papaya, heterosis has been successfully exploited to improve growth, yield-attributing traits, and fruit quality traits (Marin et al., 2006; Cardoso et al., 2014; Singh et al., 2019; Miranda et al., 2025). Moreover, F_1_ hybrids resistant to diseases such as phoma and black spot have also been developed (Vivas et al., 2016; Poltronieri et al., 2019). Similar studies on heterosis and combining ability for quality and nutritional traits have been conducted in many other horticultural crops like cauliflower (Dey et al., 2014), strawberry (Kaczmarska et al., 2017), tomato (Liu et al., 2021; Pavan et al., 2022; Mendes et al., 2026), muskmelon (Badami et al., 2020; Kaur et al., 2022; Martins et al., 2025) and pumpkin (Hosen et al., 2022). Integration of PCA with combining ability and heterosis analysis can provide a more comprehensive assessment of parental and hybrid performance for simultaneous improvement of multiple fruit quality traits. Beyond the importance of the papaya as a major fruit crop in India, it also represents a useful model for quality-oriented breeding under changing climatic conditions. The integration of heterosis, combining ability and multivariate approaches such as PCA provides a broadly applicable framework for identifying parental combinations that simultaneously improve nutritional quality, antioxidant potential and postharvest shelf life. Such breeding strategies are particularly relevant for developing climate-resilient and nutritionally enriched fruit varieties capable of meeting the increasing demand for functional foods. Based on these considerations, the present study hypothesized that genetically diverse papaya inbred lines differ significantly in their combining ability for fruit nutritional quality and shelf-life traits, and that superior parental combinations can be identified through diallel analysis for development of improved hybrids. The novelty of this study lies in the simultaneous evaluation of general combining ability, specific combining ability and heterosis for multiple nutraceutical and postharvest quality attributes, thereby providing valuable insights for the selection of elite parents and hybrid combinations in papaya breeding programs.

## 2 Materials and methods

### 2.1 Crossing of parental lines and raising of F1 hybrids

The investigation aimed to evaluate heterosis and combining ability for fruit quality traits in papaya and was carried out at the Division of Fruits and Horticultural Technology, ICAR-IARI, New Delhi. The location experiences a typical subtropical climate, characterized by high seasonal temperature variation. For the study, six inbred parental lines of papaya developed at the Indian Agricultural Research Institute, New Delhi, exhibiting diverse morphological and fruit quality traits, were used as parents. The selected parents, namely, Pune Selection 3 (PS3), Pusa Nanha (PN), P-7-2, P-7-9, P-9-5, and P-9-12, were crossed in a full diallel mating design (**Table 1**), including reciprocal crosses, resulting in 30 F_1_ hybrids. Ten plants from each selected parental line were planted in insect-proof net houses to prevent unwanted pollination. At the flowering stage, when the flowers opened slightly or the ones opening the next day were selected for making crosses. Later on, pollen from open male or hermaphroditic flowers was collected in a Petri dish for pollination. The collected pollen was then dusted over the selected female and hermaphrodite flowers. All parental lines were maintained by selfing in gynodioecious genotypes, such as P-7-2, P-7-9, P-9-5, and P-9-12, and by sibmating in dioecious genotypes, such as PS3 and PN. The seeds of F_1_ hybrids and parents were collected from ripened fruits, washed and dried in shade. The seedlings of six parents, along with thirty crosses, were raised and transplanted in a randomized block design with three replications. The plants were raised as per the recommended package of practices for papaya cultivation under the north Indian plains. The young plants were covered with polythene during the winter months to prevent low-temperature injury. For assessing the fruit quality of different cross combinations of papaya, three fruits from each replication were harvested at uniform physiological maturity and analyzed for different fruit quality and shelf-life traits like total soluble solids (TSS), total phenolic content (TPC), total flavonoid content (TFC), antioxidant activity, total carotenoids, β-carotene, lycopene, fruit firmness and shelf life of the fruits.

**Table 1:** Details of parent/inbred lines used in the diallel crossing.

Sl. no.	Parents	Descriptive features	Source	Reference fruit image
1	Pune Selection 3 (PS3)	Dioecious, round and oval shape	ICAR-IARI, New Delhi	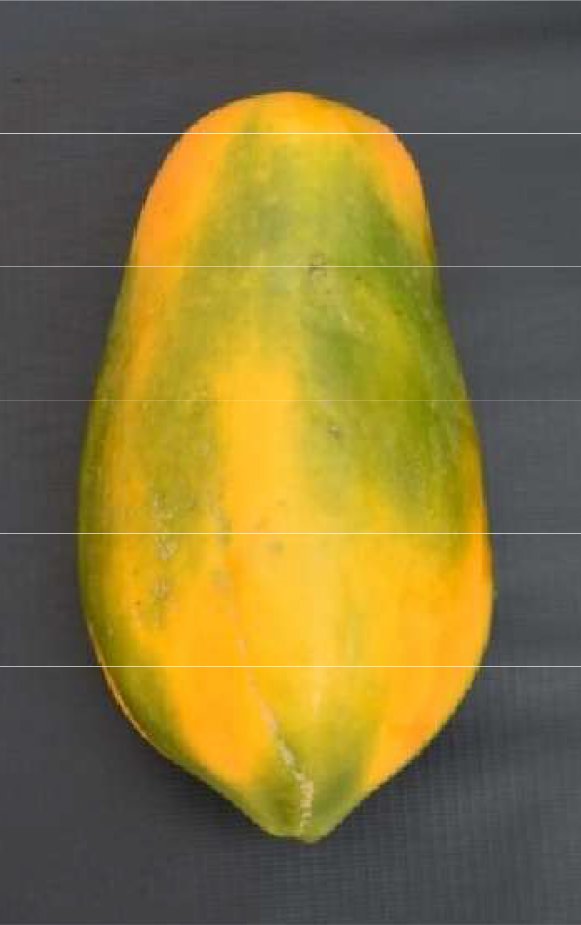
2	Pusa Nanha (PN)	Dioecious, round shape	ICAR-IARI, New Delhi	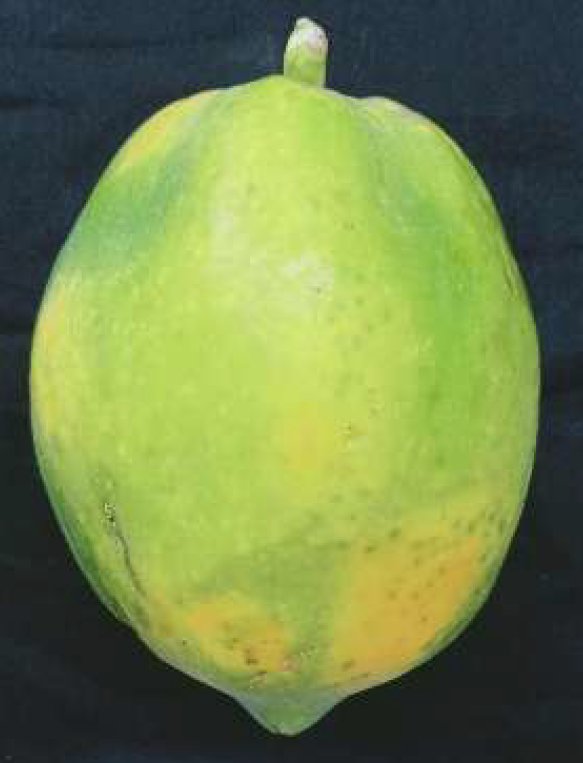
3	P-7-2	Gynodioecious, round and oval shape	ICAR-IARI, New Delhi	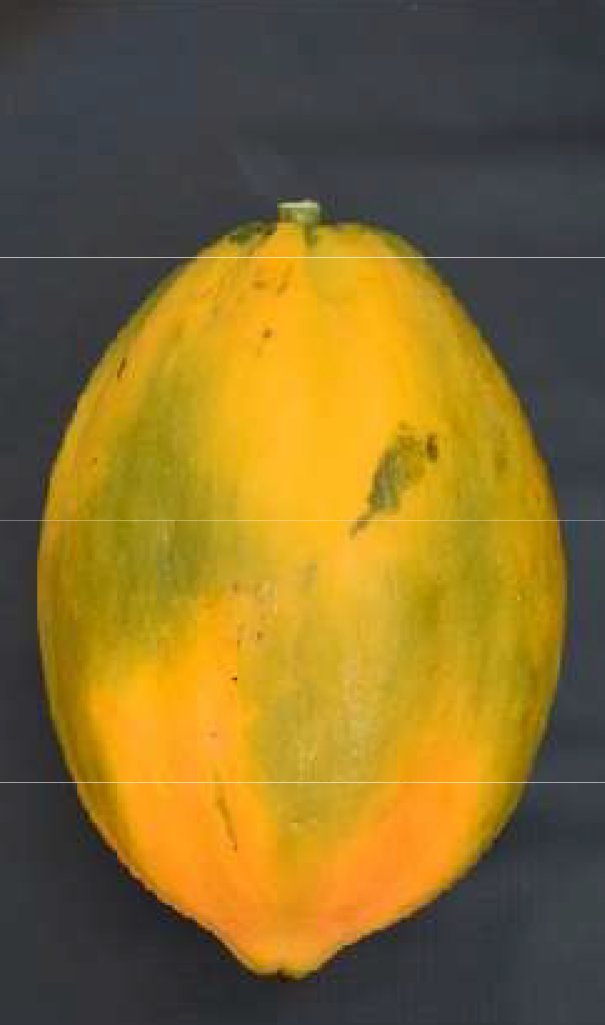
4	P-7-9	Gynodioecious, round and oval shape	ICAR-IARI, New Delhi	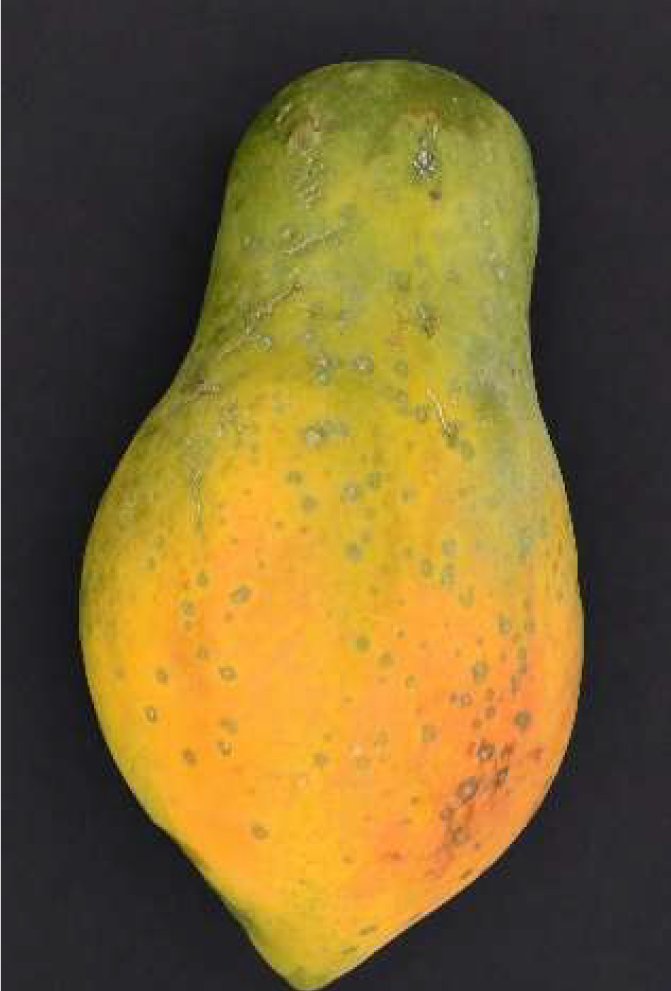
5	P-9-5	Gynodioecious, round and oval shape	ICAR-IARI, New Delhi	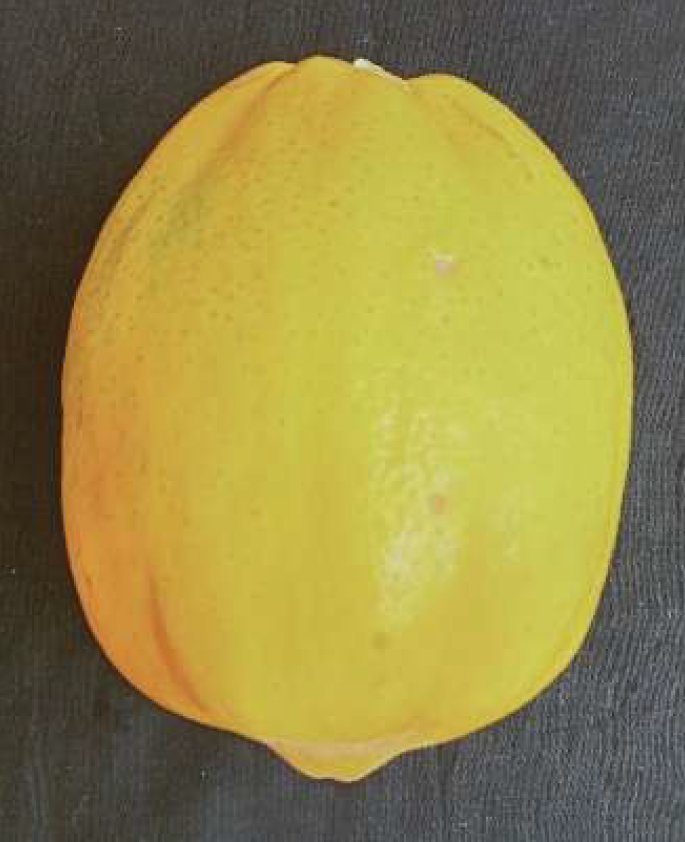
6	P-9-12	Gynodioecious, round and oval shape	ICAR-IARI, New Delhi	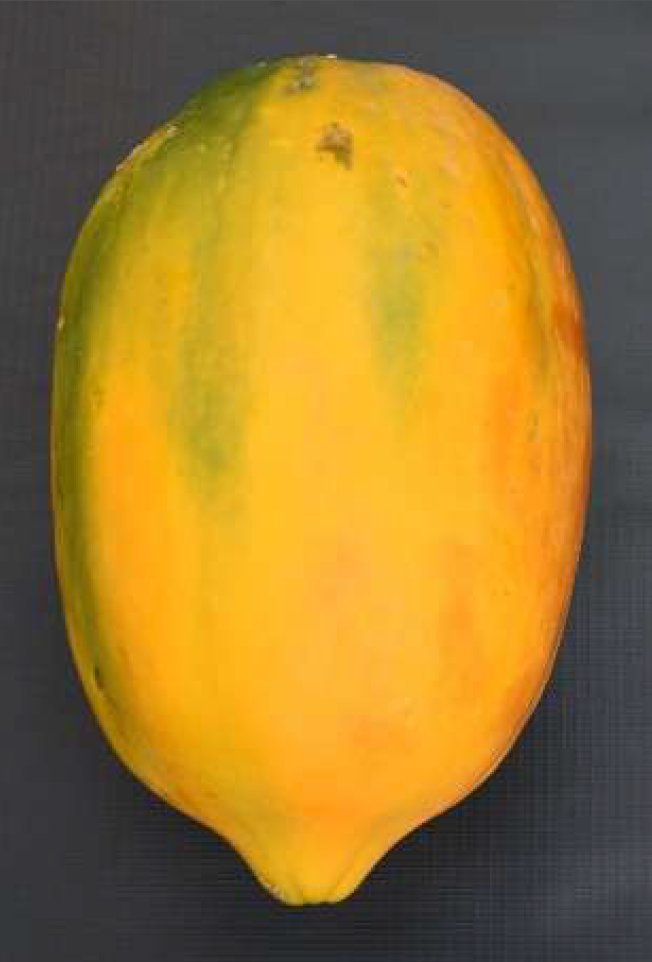

### 2.2 Data collection for fruit quality and shelf-life analysis

#### 2.2.1 Determination of total soluble solids

The TSS of papaya fruits was assessed using a hand refractometer (Atago, Japan) at 25 °C. The fruit pulp was mashed, and the juice was squeezed through a muslin cloth. A few drops of homogenized papaya juice were added to the prism of the refractometer. The obtained values were expressed in ^0^Brix.

#### 2.2.2 Total phenolic content, total flavonoid content, and antioxidant activity

For estimation of TPC, TFC and antioxidant capacity, a sample extract was prepared by crushing 10 g of papaya fruit sample in 20 mL of 80% ethanol, and the resultant mixture was then centrifuged (Sigma) for 20 minutes at 10,000 rpm. The acquired supernatant was collected and stored at 4^0^C for further analysis. The total phenolic content of the fruits was determined using the Folin-Ciocalteau reagent (FCR) method as described by Singleton et al. (1999) and absorbance was measured at 765 nm. The results were expressed as mg gallic acid equivalent/kg of fresh weight. The total flavonoid content of the fruit was assessed using the aluminum chloride colorimetric assay as demonstrated by Zhishen et al. (1999), and absorbance was recorded at 510 nm using a spectrophotometer. The values were expressed as mg catechin equivalent/kg of fresh weight. The determination of antioxidant activity through the DPPH (2,2-diphenyl-1-picrylhydrazyl) scavenging system was carried out according to the method described by Musa et al. (2011), and absorbance was measured at 517 nm using a UV-Vis double beam spectrometer (UVD-3200, Labomed, Inc., USA).

#### 2.2.3 Determination of β-carotene, lycopene and total carotenoids

A sample extract was prepared by crushing 2 g of the papaya fruit sample in 15 ml of acetone for the determination of β-carotene, lycopene, and total carotenoids content in the papaya fruits of parents and hybrids. The resultant sample was then kept in the dark overnight. β-carotene, lycopene and total carotenoids estimation was done by the method as outlined in AOAC (2006). Absorbance was recorded using a UV-VIS double-beam spectrophotometer (UVD 3200, Labomed, Inc., USA) at 452 nm, 450 nm, and 502 nm for total carotenoids, β-carotene, and lycopene, respectively, against a reagent blank (Blank- Hexane).

#### 2.2.4 Determination of fruit firmness and shelf life

The firmness of different fruits was assessed using a texture analyzer (TA + Di Texture Analyzer) at three points on each fruit: apex, middle, and base. Firmness was expressed in Kg/m^2^. On average, three ripe fruits from different papaya genotypes were stored under ambient conditions (25 ± 2^0^C and RH of 70 ± 5%) to assess their postharvest shelf life. Fruits exhibiting more than 20% physiological loss in weight, along with symptoms of over-ripening and fungal spores growing on them, were considered unfit for consumption and discarded. The days were recorded before the occurrence of the above-mentioned signs.

### 2.3 Statistical analysis

The mean performances of paternal inbred lines and hybrids were compared using the critical difference (CD) value calculated using a 5% significance level (α = 0.05). Principal Component Analysis was performed using R software version 4.4.0. Statistical analysis of the general combining ability of parents and the specific combining ability of hybrids in a full diallel mating design was done following Model I and Method III of Griffing (1956) using OPSTAT software (Sheoran et al., 1998). The mathematical model for the combination of the ability analysis is:


pijk=μ+gi+gj+sij+rk+eijk

where ij is the number of parents, k is the number of blocks or replications, Pijk is the phenotype of the ijk^th^ observation, µ is the population mean, gi (or gj) is the GCA of the i^th^ or j^th^ parent, s^ij^ is the SCA of the cross between the i^th^ female and the j^th^ male parent, rk is the effect of the k^th^ replication, and eijk is the environmental effect particular to the ijk^th^ observation.

Mid-parent heterosis (MPH) and better parent heterosis (BPH) values were calculated to quantify the percentage of positive and negative increases in F_1_ hybrids over their respective parents for the different traits under study. Mid-parent and better-parent heterosis were calculated using the formula:


$$MPH=\frac{F1-MP}{MP}$$

$$BPH=\;\frac{F1-BP}{BP}$$

Where MP and BP are the means of the mid-parent and better-parent, respectively.

## 3 Results

### 3.1 Mean performance of parents and hybrids

Assessment of the per se performance of the six parental inbred lines and their 30 hybrids revealed considerable variation for all nutritional and postharvest quality traits, indicating substantial genetic variability for quality improvement (**Table 2**). Total soluble solids (TSS) varied within a relatively narrow range (8.87^0^Brix to 12.80^0^Brix). Among the parents, P-9–12 exhibited the highest TSS (11.97^0^Brix), while the hybrid P-9–12 x P-9–5 recorded the maximum value (12.80^0^Brix), suggesting the potential of this combination for improving fruit sweetness. The nutritional traits exhibited much wider variation than TSS. Total phenolic content (TPC) and total flavonoid content (TFC) ranged from 52.87 mg/100g to 147.80 mg/100g and 16.40 mg/100g to 72.53 mg/100g, respectively. P-9–5 and P-7–9 were the superior parental lines for TPC and TFC, whereas the hybrid P-7–9 x P-9–5 recorded the highest values for both traits (147.80 mg/100g and 72.53 mg/100g, respectively). Similarly, P-9–12 exhibited the highest antioxidant activity among the parents (2.82 µmol/g), while the hybrid P-9-5 × P-7–9 showed the greatest antioxidant capacity (2.87 μmol/g). Considerable variation was also observed for carotenoid pigments. P-9–12 was the best-performing parent for β-carotene, while P-7–9 recorded the highest lycopene and total carotenoid contents. Hybridization further enhanced pigment accumulation, with P-9-12 × P-7–9 recording the highest β-carotene (3.76 mg/100 g), P-9–12 x P-9–5 exhibiting the highest lycopene (9.18 mg/100 g), and PS3 x P-9–12 producing the highest total carotenoid content (12.18 mg/100 g). Significant differences were also observed for postharvest quality traits. P-9–12 exhibited the highest fruit firmness (4.76 kg/cm^2^) and shelf life (8.33 days) among the parental lines. Among the hybrids, P-9–12 x PS3 showed the greatest fruit firmness (4.42 kg/cm^2^), whereas P-9–12 x P-9–5 recorded the longest shelf life (8.67 days). The results revealed that fruit firmness and shelf life are related traits, as the shelf life of firmer fruits is longer than that of mellow fruits at ripening. The mean performance results also showed that the hybrids outperformed the parental lines on the majority of traits. As the parents and hybrids in the study showed significant differences in papaya quality traits, this allows the utilization of genetic diversity to improve fruit quality through heterosis.

**Table 2:** Mean performance of papaya parent and hybrid genotypes for fruit quality and shelf-life traits.

Genotype	Total soluble solids (°Brix)	Total phenol content (mg/100g)	Total flavonoid content (mg/100g)	Antioxidant activity (DPPH) (µmol/g)	β-carotene (mg/100g)	Lycopene (mg/100g)	Total carotenoids (mg/100g)	Fruit firmness (kg/m^2^)	Fruit shelf life (days)
PN	8.87	60.53	20.80	2.18	1.07	4.38	7.75	2.45	4.00
PN x PS3	12.10	71.07	23.53	2.22	1.47	2.00	5.18	3.25	6.33
PN x P-7-2	11.57	58.20	28.27	2.35	1.20	4.09	7.18	2.63	4.67
PN x P-7-9	10.80	68.87	54.80	2.77	2.45	2.46	5.57	2.88	5.67
PN x P-9-5	12.50	93.40	50.47	2.41	2.22	8.12	9.58	3.04	6.00
PN x P-9-12	12.23	68.13	42.87	2.81	2.61	3.32	4.95	3.63	6.33
P-7-2	10.47	54.93	18.13	1.63	0.97	2.51	2.57	2.84	5.33
P-7–2 x PS3	11.37	57.27	26.80	2.05	1.69	3.15	4.03	3.65	6.33
P-7–2 x PN	9.67	62.20	37.40	2.31	1.52	4.21	4.96	2.54	3.67
P-7–2 x P-7-9	11.03	74.73	70.80	2.67	2.85	1.88	2.87	3.07	6.67
P-7–2 x P-9-5	11.90	99.27	48.27	2.58	2.14	7.85	7.51	3.36	7.00
P-7–2 x P-9-12	12.30	72.07	28.40	2.74	3.04	5.01	5.41	3.83	7.67
P-7-9	11.40	99.67	58.53	2.96	3.27	7.09	8.56	3.63	8.00
P-7–9 x PS3	12.07	78.73	27.13	2.03	2.30	3.65	5.72	3.90	6.33
P-7–9 x PN	10.13	89.80	33.80	2.11	2.25	5.28	8.22	2.76	5.33
P-7–9 x P-7-2	11.10	69.93	32.93	2.54	2.02	2.61	4.30	3.02	5.00
P-7–9 x P-9-5	11.60	147.80	72.53	2.68	2.92	7.23	8.96	3.53	7.33
P-7–9 x P-9-12	12.57	66.67	26.87	2.63	3.54	8.97	9.23	4.07	7.67
P-9-5	11.47	127.53	34.27	3.01	1.83	3.00	6.58	3.91	7.00
P-9–5 x PS3	9.57	107.13	27.13	2.22	1.99	5.18	7.74	3.97	5.67
P-9–5 x PN	9.27	115.73	45.73	2.11	1.88	6.08	8.43	2.94	5.00
P-9–5 x P-7-2	10.87	85.47	37.20	2.25	1.62	2.84	5.27	3.19	5.33
P-9–5 x P-7-9	11.33	137.60	69.87	2.87	2.70	5.85	7.99	3.69	7.00
P-9–5 x P-9-12	12.20	95.07	42.07	2.74	2.96	7.31	7.02	4.11	7.67
P-9-12	11.97	74.33	34.27	2.82	3.62	6.75	7.22	4.76	8.33
P-9–12 x PS3	10.47	75.33	32.67	2.37	2.49	2.96	7.44	4.42	6.33
P-9–12 x PN	10.50	80.73	45.73	2.41	2.30	8.07	8.54	3.38	6.00
P-9–12 x P-7-2	11.37	67.33	27.13	2.51	2.13	5.74	7.47	3.57	6.67
P-9–12 x P-7-9	12.30	94.73	69.87	2.68	3.76	4.69	6.27	3.85	7.67
P-9–12 x P-9-5	12.80	120.27	42.07	2.84	3.17	9.18	10.20	4.07	8.67
PS3	10.87	52.87	16.40	1.90	1.61	6.15	4.90	4.08	7.33
PS3 x PN	9.97	54.93	24.27	2.40	1.83	5.15	6.82	3.07	5.33
PS3 x P-7-2	10.13	40.53	24.13	2.18	1.62	3.68	9.00	3.28	7.33
PS3 x P-7-9	11.67	97.93	31.40	2.52	2.99	5.50	11.28	3.55	8.00
PS3 x P-9-5	12.40	106.47	28.67	2.84	2.59	4.27	6.16	3.94	7.33
PS3 x P-9-12	11.13	74.07	20.60	2.69	3.13	7.01	12.18	4.29	8.67
C.D. (*p* = 0.05)	1.10	7.01	8.14	0.08	0.06	0.73	0.42	0.16	1.20
SE (m)	0.39	2.48	2.88	0.03	0.02	0.26	0.15	0.06	0.43
SE (d)	0.55	3.51	4.07	0.04	0.03	0.36	0.21	0.08	0.60
C.V.	6.01	5.15	13.25	2.06	1.52	8.74	3.64	2.81	11.29

### 3.2 General combining ability analysis

General combining ability (GCA) analysis revealed significant differences among the six parental lines for all nutritional and postharvest quality traits, indicating substantial variation in their ability to transmit favorable alleles to the progeny (**Table 3**). P-9–12 emerged as the best general combiner for most of the evaluated traits, exhibiting significant positive GCA effects for TSS (0.67), antioxidant activity (0.20), β-carotene (0.67), lycopene (1.43), total carotenoids (0.98), fruit firmness (0.35) and shelf life (1.04). In contrast, P-9–5 was the superior general combiner for total phenolic content (28.95) and total flavonoid content (10.01) and also showed favorable GCA effects for antioxidant activity, carotenoid pigments, fruit firmness, and shelf life. P-7–9 also exhibited positive GCA effects for phenolic and flavonoid contents and β-carotene, highlighting its potential for improving nutraceutical quality. Conversely, P-7–2 consistently exhibited significant negative GCA effects for most quality traits, including TSS, antioxidant activity, carotenoid pigments, fruit firmness, and shelf life, indicating its limited usefulness as a donor for quality improvement. PN and PS3 also displayed predominantly negative GCA effects, although PS3 contributed positively to total carotenoid content. Overall, the GCA analysis identified P-9–12 as the most promising parent for improving fruit quality and postharvest traits, whereas P-9–5 and P-7–9 emerged as valuable donors for enhancing nutraceutical attributes, particularly phenolic and flavonoid contents.

**Table 3:** General combining ability effects of the parents for fruit quality and shelf-life traits.

Parents	TSS	Total phenolics	Total flavonoids	Antioxidant activity	Beta carotene	Lycopene	Total carotenoids	Fruit firmness	Shelf life
Pune Selection 3	-0.29	-8.65	-8.89	-0.05	-0.15	0.05	0.30	-0.21	-0.44
Pusa Nanha	-0.09	-11.32	-6.63	-0.14	-0.43	-1.22	-0.60	-0.04	-0.44
P-7-2	-0.55	-11.07	-1.91	-0.19	-0.41	-0.42	-0.89	-0.30	-0.55
P-7-9	0.09	1.37	8.92	0.05	0.20	-0.43	-0.22	-0.08	-0.27
P-9-5	0.17	28.95	10.01	0.13	0.12	0.60	0.43	0.28	0.65
P-9-12	0.67	0.71	-1.50	0.20	0.67	1.43	0.98	0.35	1.04
S.E. (g_i_)	0.10	0.65	0.65	0.01	0.01	0.07	0.04	0.02	0.11
S.E. (g_i_-g_j_)	0.28	1.75	1.74	0.02	0.01	0.18	0.10	0.04	0.30
CD at 5%	0.56	3.50	3.48	0.04	0.02	0.36	0.20	0.08	0.60
CD at 1%	0.74	4.66	4.63	0.05	0.03	0.48	0.27	0.11	0.80

### 3.3 Specific combining ability analysis

Specific combining ability (SCA) analysis of the 30 hybrid combinations revealed substantial variation for all nutritional and postharvest quality traits, indicating the importance of non-additive gene action in the expression of these characteristics (**Table 4**). Significant positive SCA effects were observed for several hybrid combinations; however, no single cross was superior for all the evaluated traits. For TSS, PN × P-7–2 exhibited the highest positive SCA effect (0.89), followed by PS3 x P-9-5 (0.83). The hybrids PS3 × P-7–9 and PS3 × P-9–5 consistently showed favorable SCA effects for nutraceutical traits, with PS3 × P-7–9 recording the highest positive effects for total phenolic content (22.71), β-carotene (0.75), and total carotenoids (2.81), while PS3 × P-9–5 exhibited the highest SCA effects for antioxidant activity (0.37) and among the highest for total phenolic content. For total flavonoid content, P-9–12 x P-9–5 emerged as the best-performing cross (16.17), whereas PN × P-9–5 recorded the highest SCA effect for lycopene (2.18). Regarding postharvest quality, P-9-5 × PS3 exhibited the highest SCA effect for fruit firmness (0.86), while P-7–2 x PS3 was the best specific combiner for shelf life (1.67). Overall, the SCA analysis demonstrated that different parental combinations contributed favorably to different quality attributes, emphasizing the importance of exploiting non-additive genetic effects for the development of superior papaya hybrids.

**Table 4:** Estimates of specific combining ability effects in cross combinations for fruit quality and shelf life traits.

Genotype	TSS	Total phenolics	Total flavonoids	Antioxidant activity (DPPH)	Beta carotene	Lycopene	Total carotenoids	Firmness	Shelf life
PS3 x PS3	-0.29	-8.65	-8.89	-0.05	-0.15	0.05	0.30	-0.21	-0.44
PS3 x PN	-1.43	-5.67	0.79	0.01	-0.30	0.85	0.56	-0.21	0.19
PS3 x P-7-2	-0.08	-15.92	-5.33	-0.33	-0.47	-1.62	-0.65	0.13	0.80
PS3 x P-7-9	0.51	22.71	7.67	0.28	0.75	1.59	2.81	0.18	0.52
PS3 x P-9-5	0.83	13.33	0.08	0.37	-0.08	-2.10	-1.39	0.32	0.44
PS3 x P-9-12	-0.05	-1.24	-0.11	0.14	0.53	0.32	1.39	0.21	-0.29
PN x PN	-0.09	-11.32	-6.63	-0.14	-0.43	-1.22	-0.60	-0.04	-0.44
PN x PS3	-0.55	2.80	-1.73	-0.11	-0.38	-0.39	0.47	-0.20	-0.50
PN x P-7-2	0.89	-3.26	-1.19	0.05	-0.04	0.17	0.07	0.06	-0.54
PN x P-7-9	0.21	0.38	2.38	0.02	0.27	-0.38	-0.56	0.14	0.35
PN x P-9-5	-0.27	-0.74	-3.04	-0.15	0.09	2.18	1.80	0.14	-0.07
PN x P-9-12	-0.45	-1.04	1.53	0.05	-0.02	-2.16	-1.22	-0.36	-0.62
P-7–2 x P-7-2	-0.55	-11.07	-1.91	-0.19	-0.41	-0.42	-0.89	-0.30	-0.55
P-7–2 x PS3	0.17	7.20	-3.00	-0.28	-0.32	-0.59	-3.22	0.50	1.67
P-7–2 x PN	-0.10	-0.47	-0.73	-0.15	0.24	-0.47	-1.58	0.68	1.33
P-7–2 x P-7-9	-0.17	8.59	13.99	0.06	0.43	-0.66	-0.38	-0.15	-0.70
P-7–2 x P-9-5	-0.26	6.25	-5.33	-0.07	-0.02	1.70	1.40	-0.03	0.05
P-7–2 x P-9-12	0.07	3.38	-6.09	0.09	0.09	0.45	-0.15	0.13	0.49
P-7–9 x P-7-9	0.09	1.37	8.92	0.05	0.20	-0.43	-0.22	-0.08	-0.27
P-7–9 x PS3	-0.13	0.87	13.57	0.22	0.14	0.80	-1.36	0.52	0.67
P-7–9 x PN	0.63	4.93	-12.87	-0.37	-0.07	0.59	0.08	0.45	-0.50
P-7–9 x P-7-2	-0.45	7.53	-12.53	-0.28	-0.30	1.70	2.68	-0.04	0.00
P-7–9 x P-9-5	-0.25	2.95	-2.07	-0.19	-0.38	-0.22	-0.12	-0.08	-0.07
P-7–9 x P-9-12	-0.01	-18.45	-4.06	-0.15	-0.36	1.27	0.56	0.06	0.55
P-9–5 x P-9-5	0.17	28.95	10.01	0.13	0.12	0.60	0.43	0.28	0.65
P-9–5 x PS3	-0.47	10.53	9.80	0.09	-0.38	-0.64	0.21	0.86	1.17
P-9–5 x PN	-1.47	6.87	-11.73	-0.10	-0.12	-1.47	-0.92	0.53	-0.33
P-9–5 x P-7-2	-1.32	8.23	-8.23	-0.24	-0.13	-0.88	0.46	-0.07	-0.67
P-9–5 x P-7-9	-0.37	-31.17	-21.20	-0.22	-0.65	-2.20	-1.85	-0.06	-0.17
P-9–5 x P-9-12	0.19	-18.13	-2.74	-0.09	0.25	-1.12	-1.80	-0.13	-0.20
P-9–12 x P-9-12	0.67	0.71	-1.50	0.20	0.67	1.43	0.98	0.35	1.04
P-9–12 x PS3	0.42	0.13	6.17	0.06	0.25	-0.13	-2.48	0.23	0.50
P-9–12 x PN	-0.88	3.60	-12.20	-0.22	-0.06	-0.18	1.25	-0.38	-1.17
P-9–12 x P-7-2	-0.90	4.33	-0.63	-0.17	-0.37	1.53	1.57	-0.40	-0.17
P-9–12 x P-7-9	-0.60	0.33	-1.43	-0.06	-0.71	-1.62	-0.88	-0.28	0.17
P-9–12 x P-9-5	0.05	-0.17	16.17	-0.03	0.40	-1.31	-0.37	-0.06	-0.67
SE (Sii)	0.33	2.07	2.05	0.02	0.02	0.21	0.12	0.05	0.35
SE (Sii-Sij)	0.45	2.87	2.84	0.03	0.02	0.30	0.17	0.07	0.49
SE (Sij)	0.23	1.49	1.48	0.02	0.01	0.15	0.09	0.03	0.26
SE (Sij-Sik)	0.36	2.27	2.25	0.03	0.02	0.23	0.14	0.05	0.39
SE (Sij-Skl)	0.32	2.03	2.01	0.02	0.02	0.21	0.12	0.05	0.35
C.D. (*p* = 0.05)	0.64	4.06	4.02	0.04	0.04	0.42	0.24	0.10	0.70

### 3.4 Principal component analysis of parents and hybrids

PCA based on the correlation matrix revealed substantial multivariate variation among the six inbred lines and their hybrid combinations for the fruit quality and nutritional value traits. The first three principal components had eigenvalues >1 and collectively explained 79.60% of the total variation, with PC1, PC2 and PC3 contributing 48.50, 16.30 and 14.80%, respectively (**Table 5**, **Figure 1**). PC1 showed a strong positive association with β-carotene (r=0.895), shelf life (r=0.856), antioxidant activity (r=0.772), fruit firmness (r=0.706), and TSS (r=0.678). PC2 was primarily associated with total flavonoid content (r=0.790) and total phenol content (r=0.563), whereas PC3 exhibited a strong association with total carotenoids (r=0.764) and lycopene (r=0.620) (**Table 6**). Among the genotypes, P-9–12 x P-9–5 recorded the highest PC1 score (1.880), while P-7–9 x P-9–5 recorded high PC1 and PC2 scores (1.323 and 1.996, respectively). PS3 x P-9–12 recorded a relatively high PC1 score (1.152) and the highest PC3 score (1.434) (**Figure 2**).

**Table 5:** Eigenvalues, percentage contribution and cumulative contribution of principal components.

Principal component	Eigen values	Variance (%)	Cumulative variance (%)
PC1	4.37	48.50	48.50
PC2	1.47	16.30	64.80
PC3	1.33	14.80	79.60
PC4	0.53	5.90	85.50
PC5	0.47	5.20	90.70
PC6	0.39	4.30	95.00
PC7	0.23	2.60	97.50
PC8	0.14	1.50	99.10
PC9	0.08	0.90	100.00

Principal components with eigenvalues >1 were considered significant according to Kaiser’s criterion.

**Figure 1:**
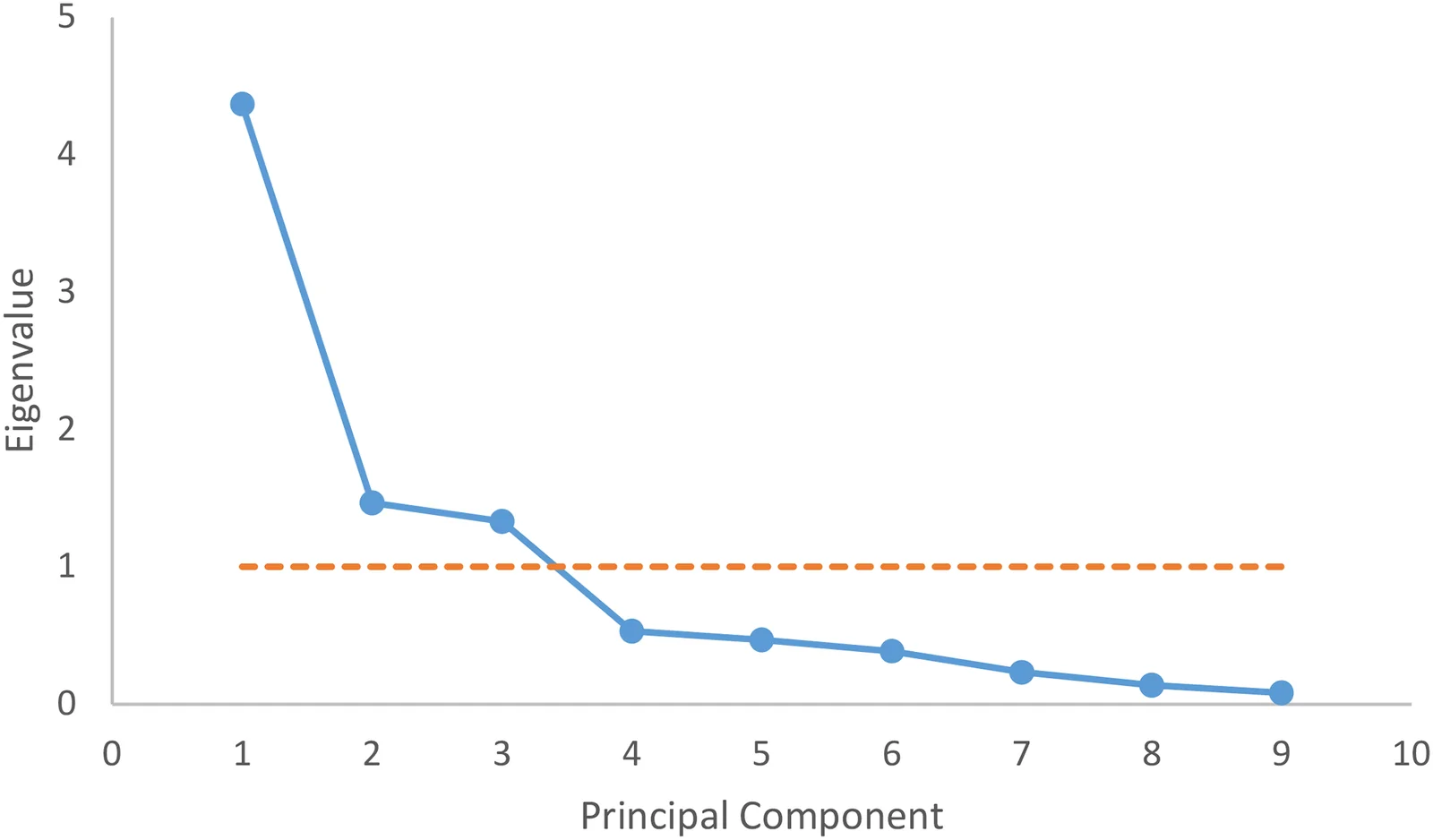
Scree plot showing the eigenvalues and percentage of variance explained by each principal component (PC). The dashed orange line indicates the Kaiser criterion (eigenvalue = 1).

**Table 6:** Correlation of fruit quality and nutritional value traits with first three principal components.

Traits	PC1	PC2	PC3
Total Soluble Solids (°Brix)	0.678	-0.224	-0.429
Total phenol content (mg/100g)	0.600	0.563	0.145
Total flavonoid content (mg/100g)	0.464	0.790	-0.211
Antioxidant activity (DPPH) (µmol/g)	0.772	0.253	-0.253
β-carotene (mg/100g)	0.895	-0.013	-0.142
Lycopene (mg/100g)	0.639	-0.053	0.620
Total carotenoids (mg/100g)	0.543	-0.035	0.764
Fruit firmness (kg/m^2^)	0.706	-0.521	-0.141
Fruit shelf life (days)	0.856	-0.369	-0.101

**Figure 2:**
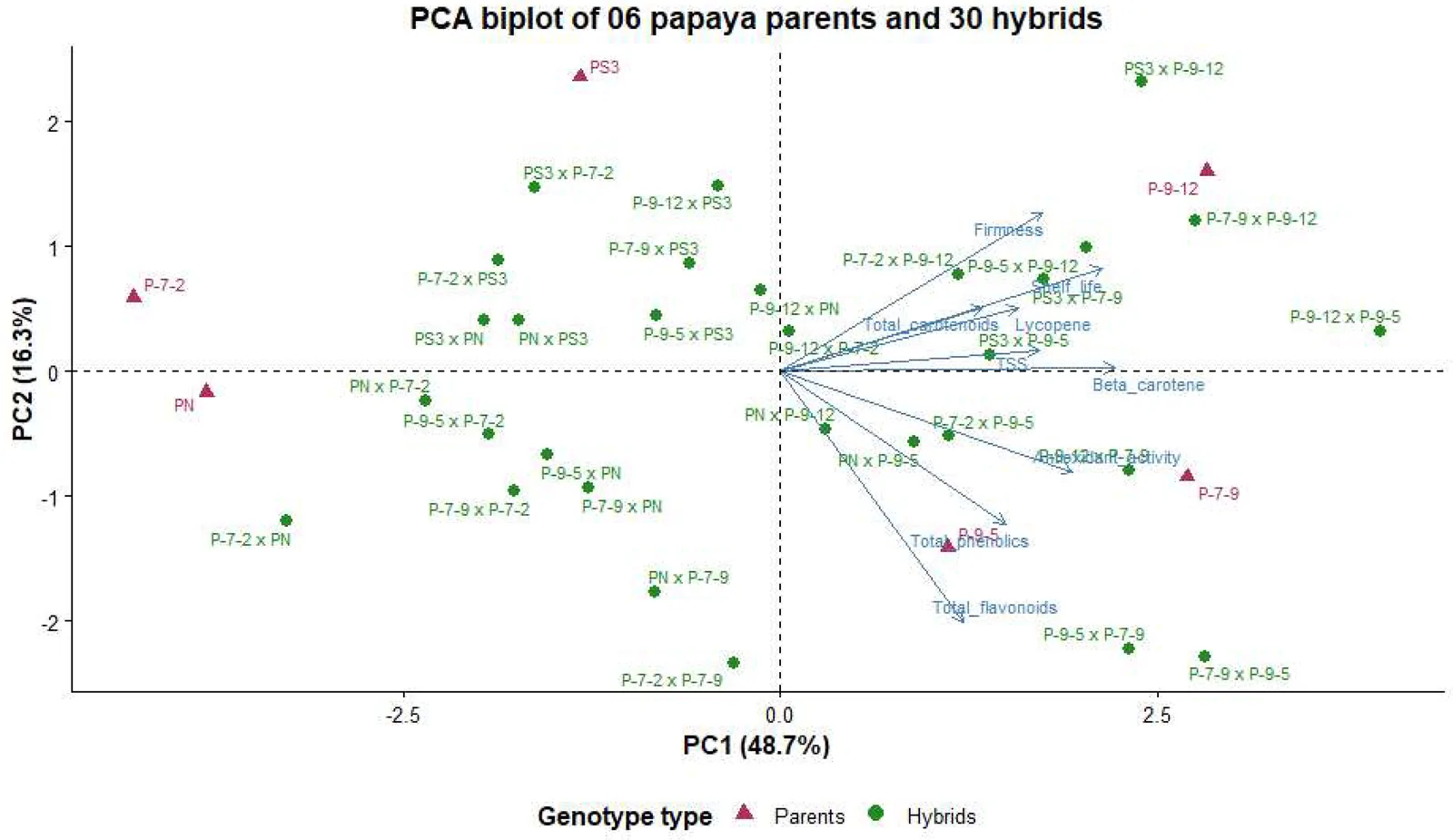
PCA biplot showing the distribution of papaya parents and hybrids and the contribution of fruit quality and nutritional traits to the first two principal components (PC1 = 48.7%, PC2 = 16.3%).

### 3.5 Nature and magnitude of mid-parent and better-parent heterosis

Heterosis analysis revealed considerable variation among the 30 hybrid combinations for all nutritional and postharvest quality traits, demonstrating the potential of hybrid breeding for improving papaya fruit quality (**Figures 3**–**11**). The magnitude of mid-parent heterosis was generally low to moderate for most traits but was substantially higher for total flavonoid content (-33.70 to 92.28%), lycopene (-62.09 to 185.86%), and total carotenoids (-48.35 to 141.19%), indicating greater scope for exploiting hybrid vigor for these nutraceutical attributes. In contrast, fruit firmness exhibited a narrow range of heterosis (-9.14 to 5.50%), and none of the hybrids expressed positive heterobeltiosis for this trait, suggesting limited non-additive genetic effects. For fruit quality traits, PN × PS3 and PN × P-7–2 exhibited the highest positive heterosis for TSS. P-7-9 × P-9–5 recorded the highest mid-parent heterosis for total phenolic content, whereas P-7-2 × PN showed the highest heterobeltiosis for total flavonoid content and β-carotene. Similarly, PN × P-7–2 and PS3 × P-7–2 exhibited the highest mid-parent and better-parent heterosis, respectively, for antioxidant activity. The hybrid P-7–2 x P-9–5 consistently recorded the highest heterotic response for both lycopene and total carotenoid contents, highlighting its potential for enhancing fruit pigmentation and nutraceutical quality. Among the postharvest traits, P-7-2 × PS3 exhibited the highest positive heterosis for shelf life under ambient storage, whereas no hybrid surpassed its better parent for fruit firmness. Overall, the heterosis analysis identified several promising hybrid combinations with trait-specific superiority, indicating that different crosses should be targeted for improving individual fruit quality attributes. The absence of positive heterobeltiosis for fruit firmness further suggests that this trait is predominantly influenced by additive genetic effects and may be more effectively improved through parental selection than through exploitation of heterosis.

**Figure 3:**
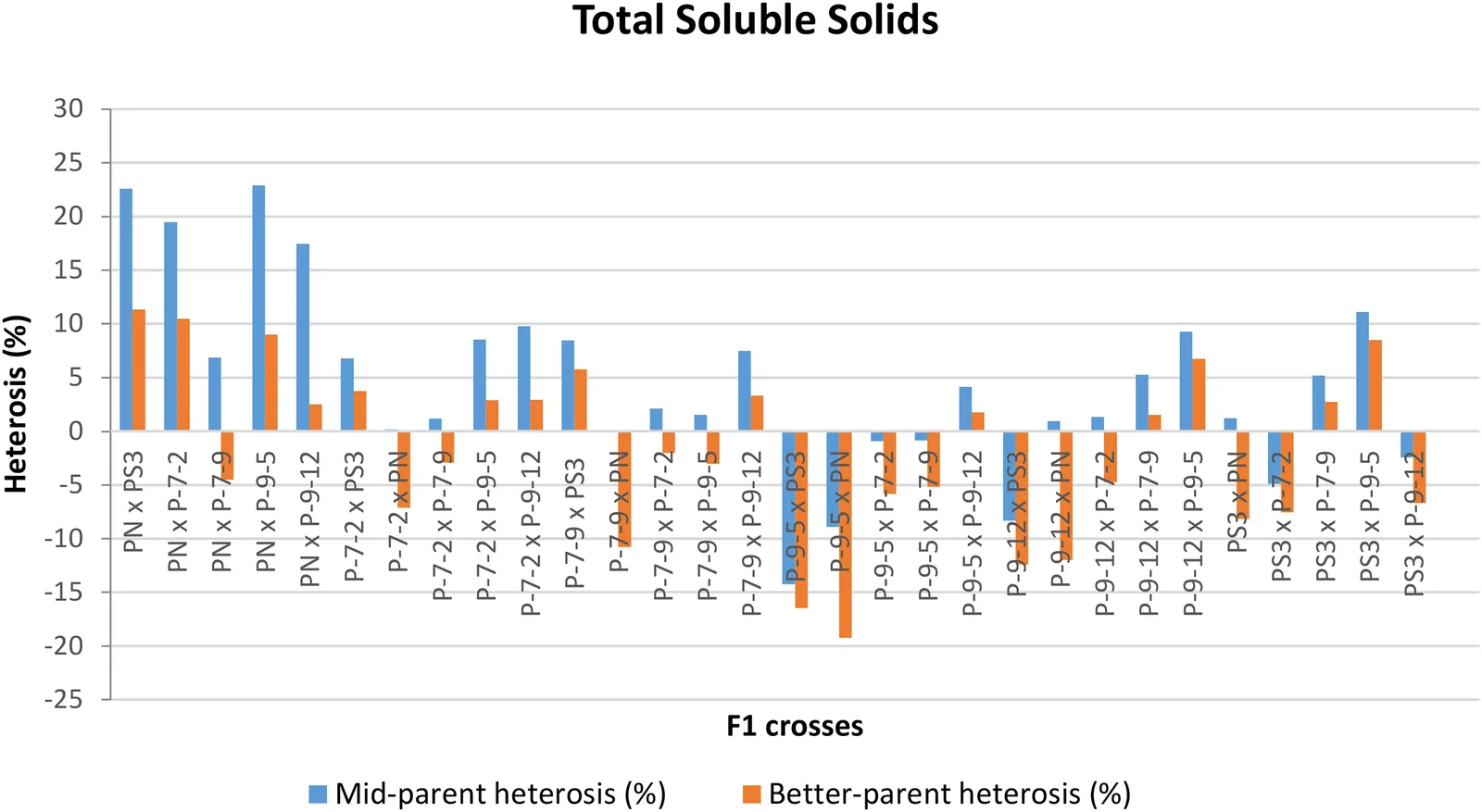
Mid-parent heterosis and better parent heterosis values in 30 F_1_ hybrids of papaya for Total Soluble Solids (TSS).

**Figure 4:**
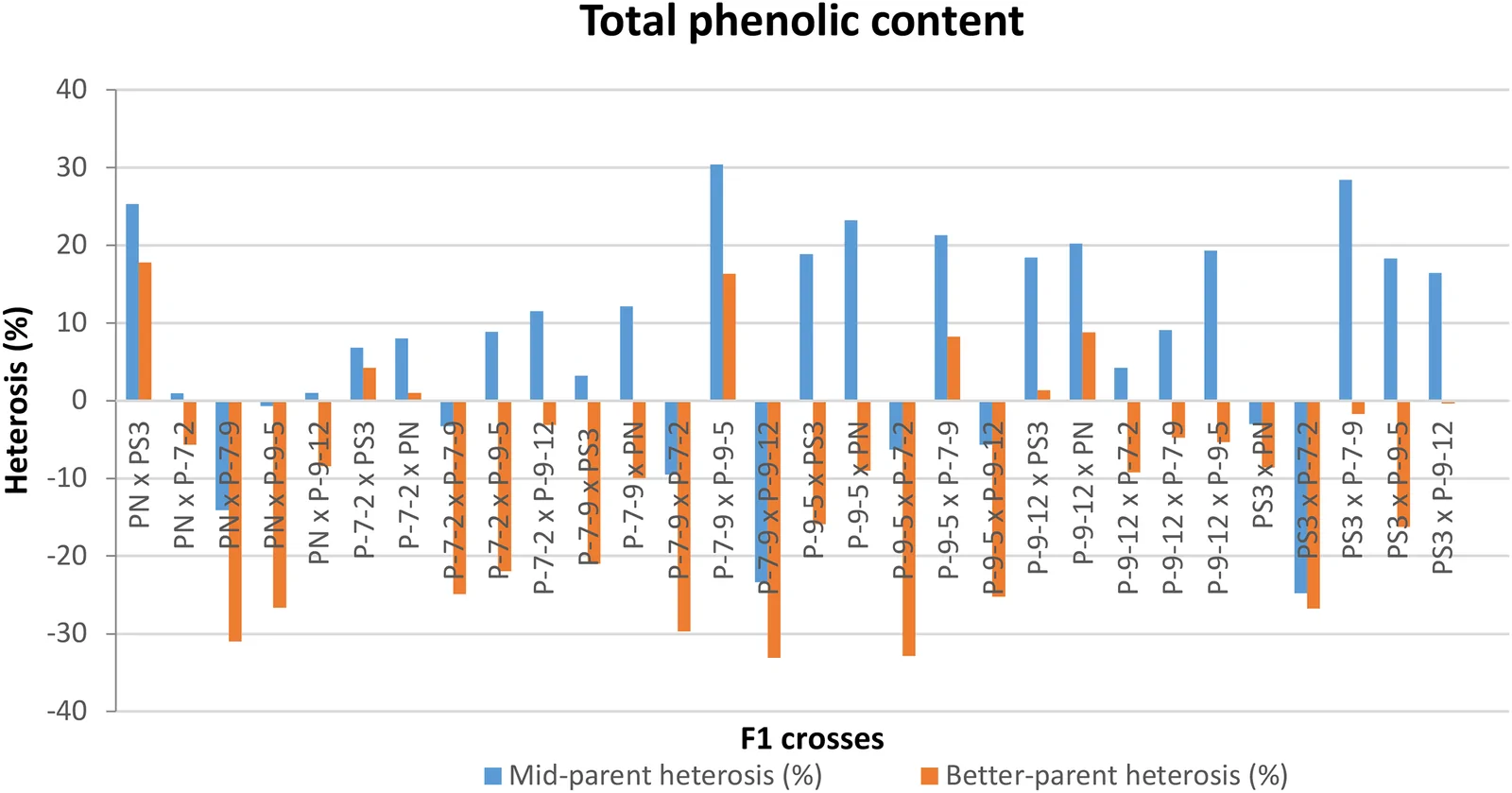
Mid-parent heterosis and better parent heterosis values in 30 F_1_ hybrids of papaya for Total Phenolic Content (TPC).

**Figure 5:**
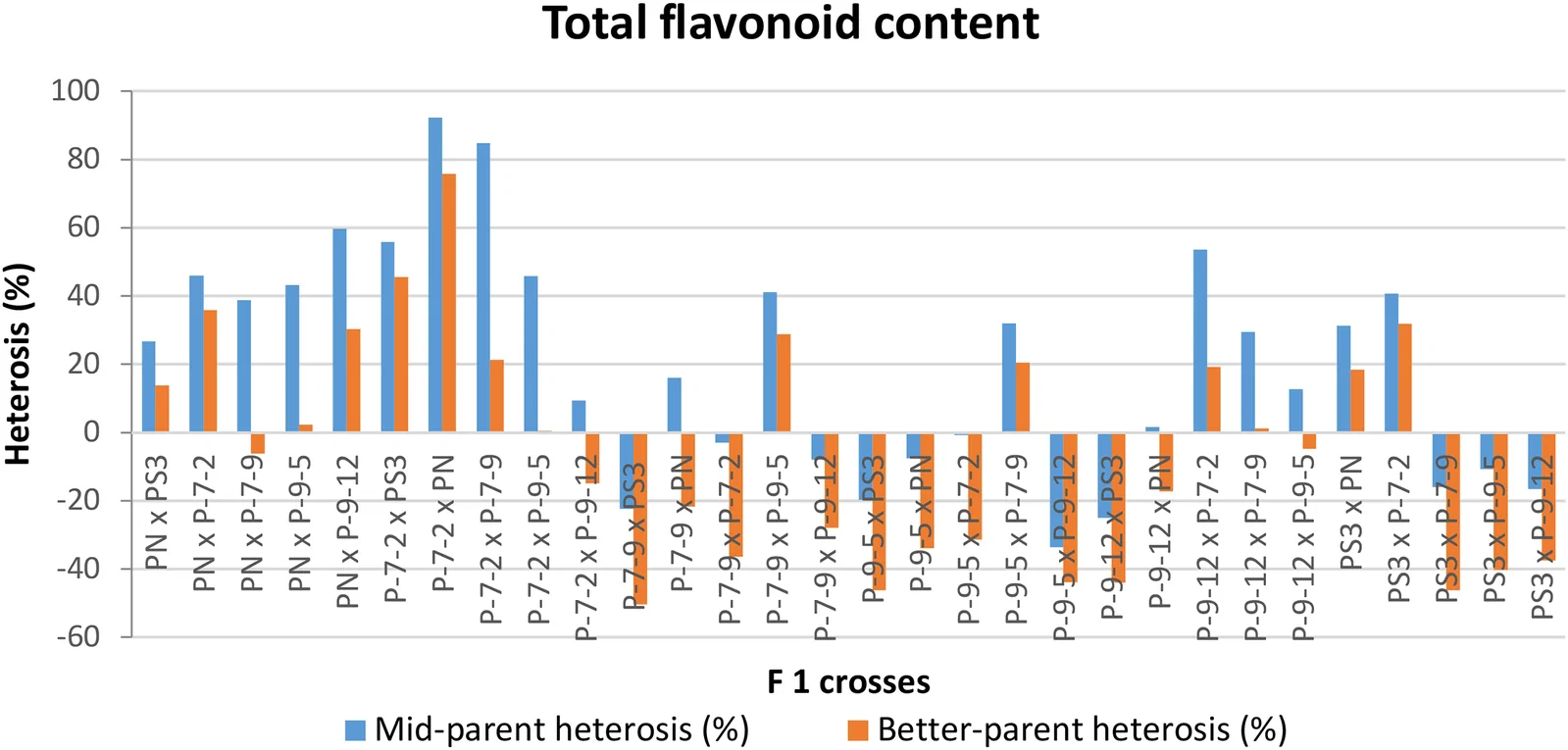
Mid-parent heterosis and better parent heterosis values in 30 F_1_ hybrids of papaya for Total flavonoid content (TFC).

**Figure 6:**
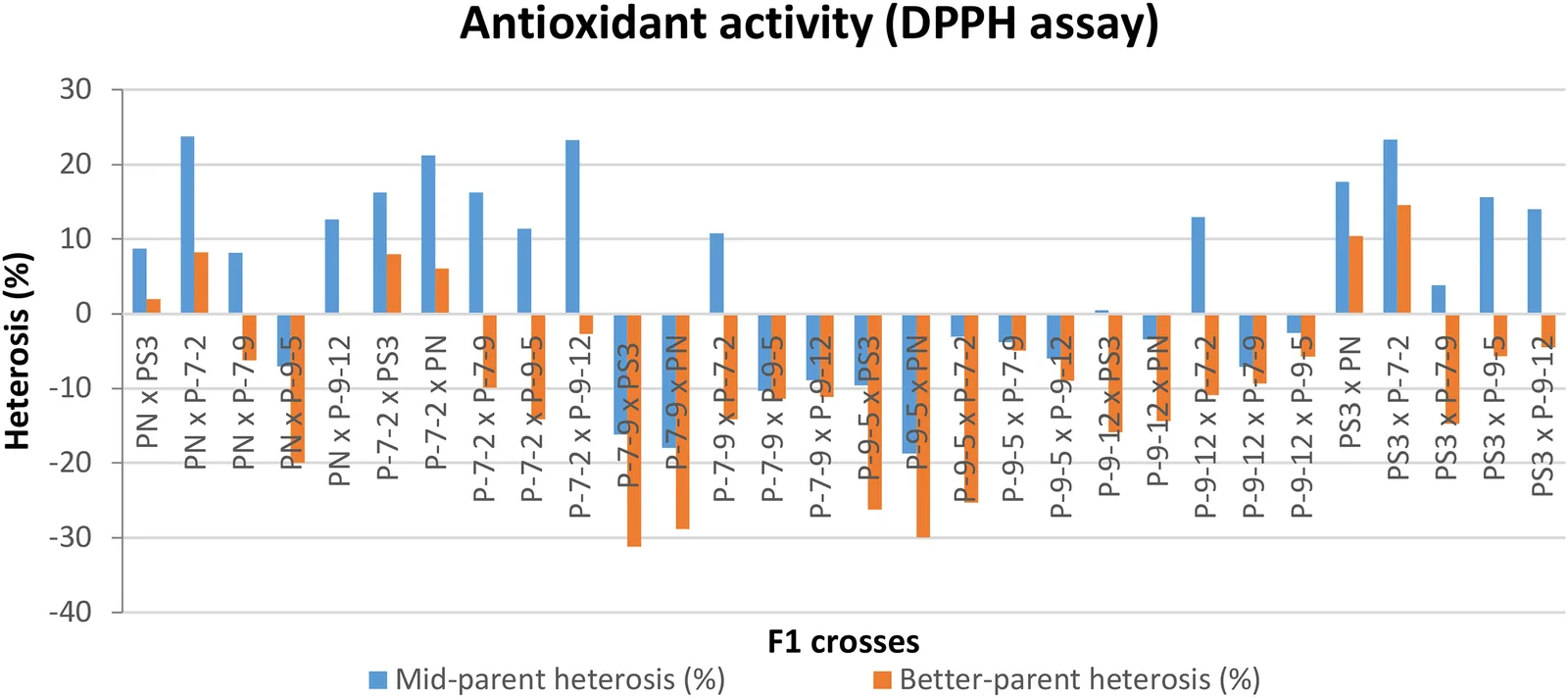
Mid-parent heterosis and better parent heterosis values in 30 F_1_ hybrids of papaya for antioxidant activity.

**Figure 7:**
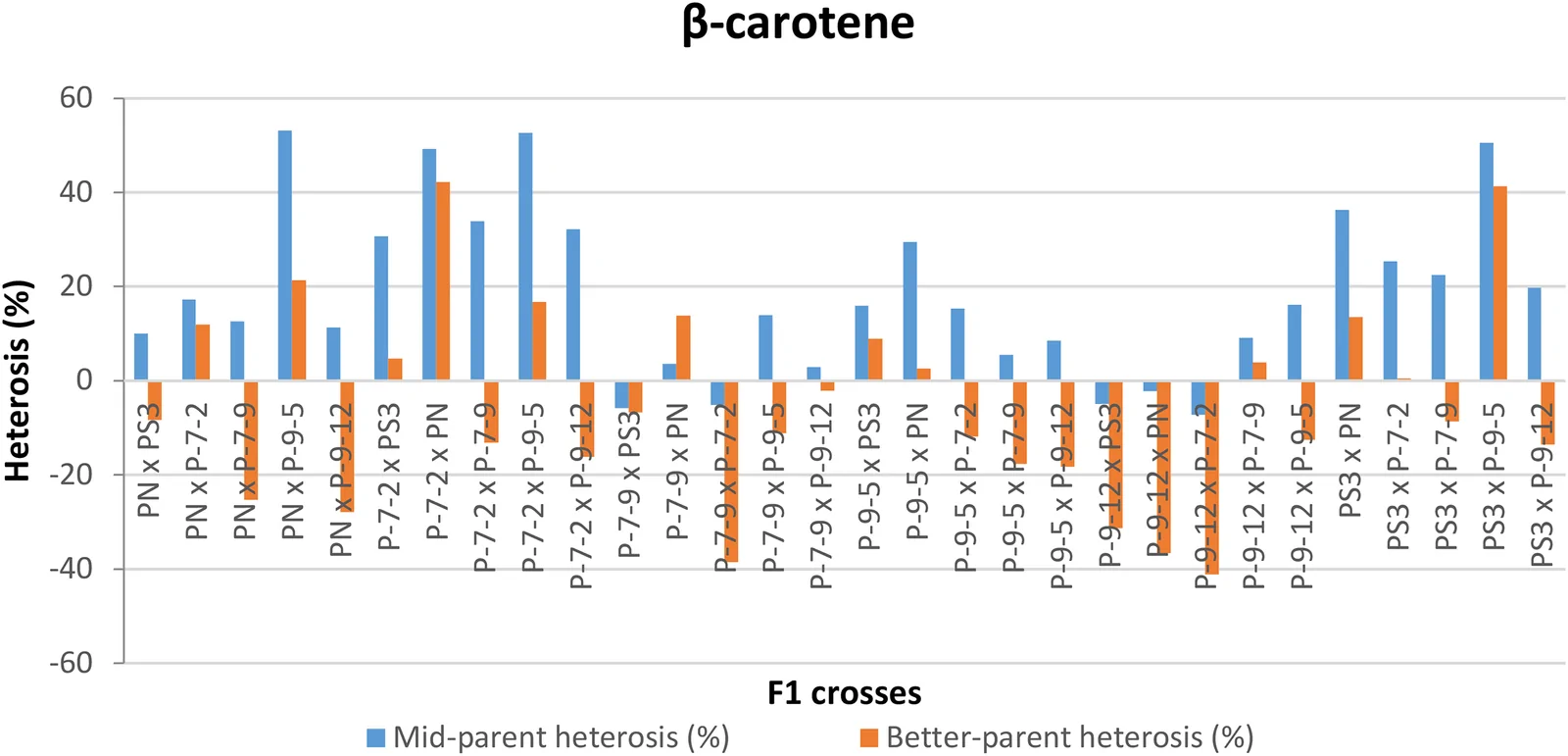
Mid-parent heterosis and better parent heterosis values in 30 F_1_ hybrids of papaya for β-carotene content.

**Figure 8:**
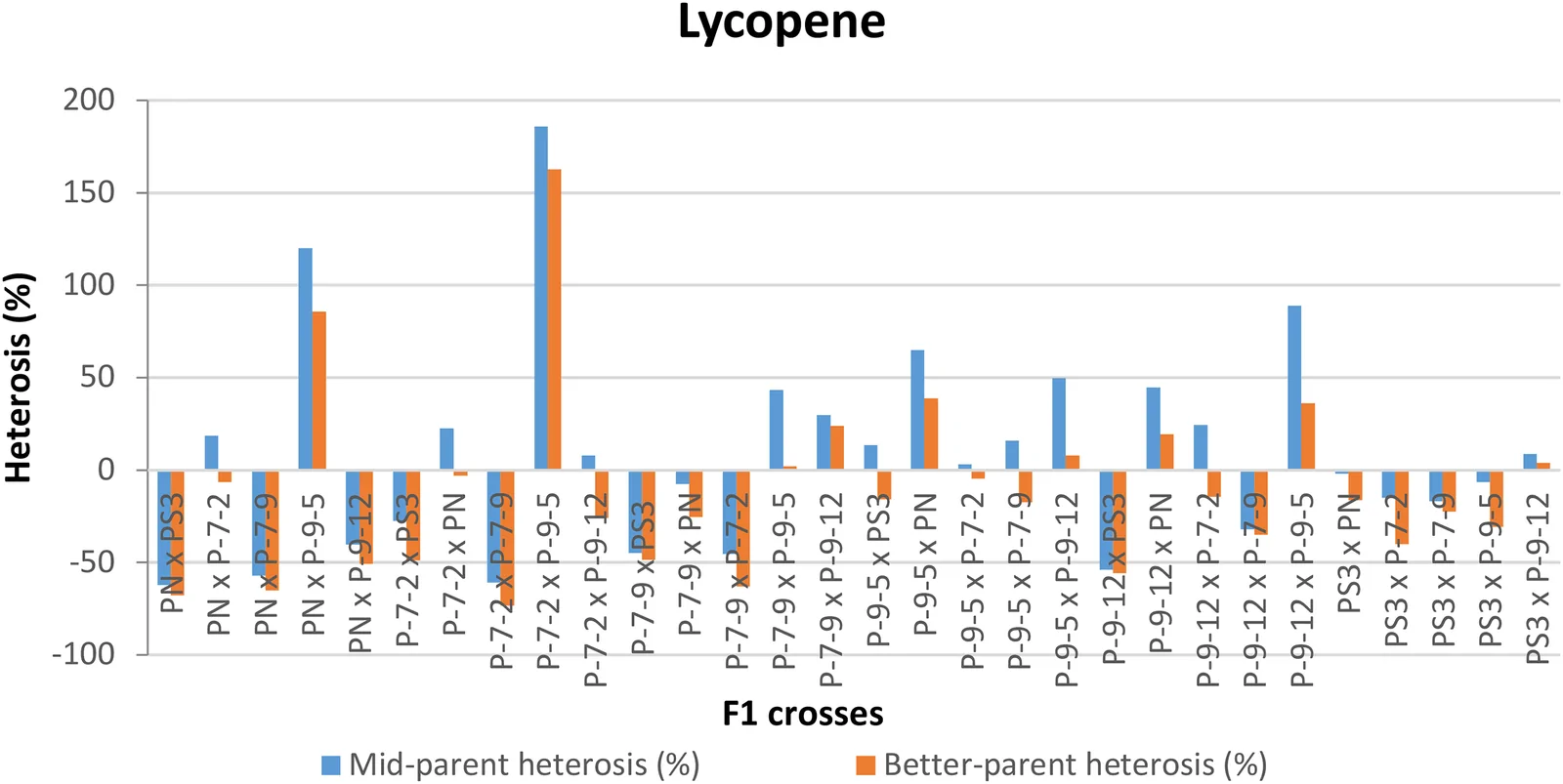
Mid-parent heterosis and better parent heterosis values in 30 F_1_ hybrids of papaya for lycopene content.

**Figure 9:**
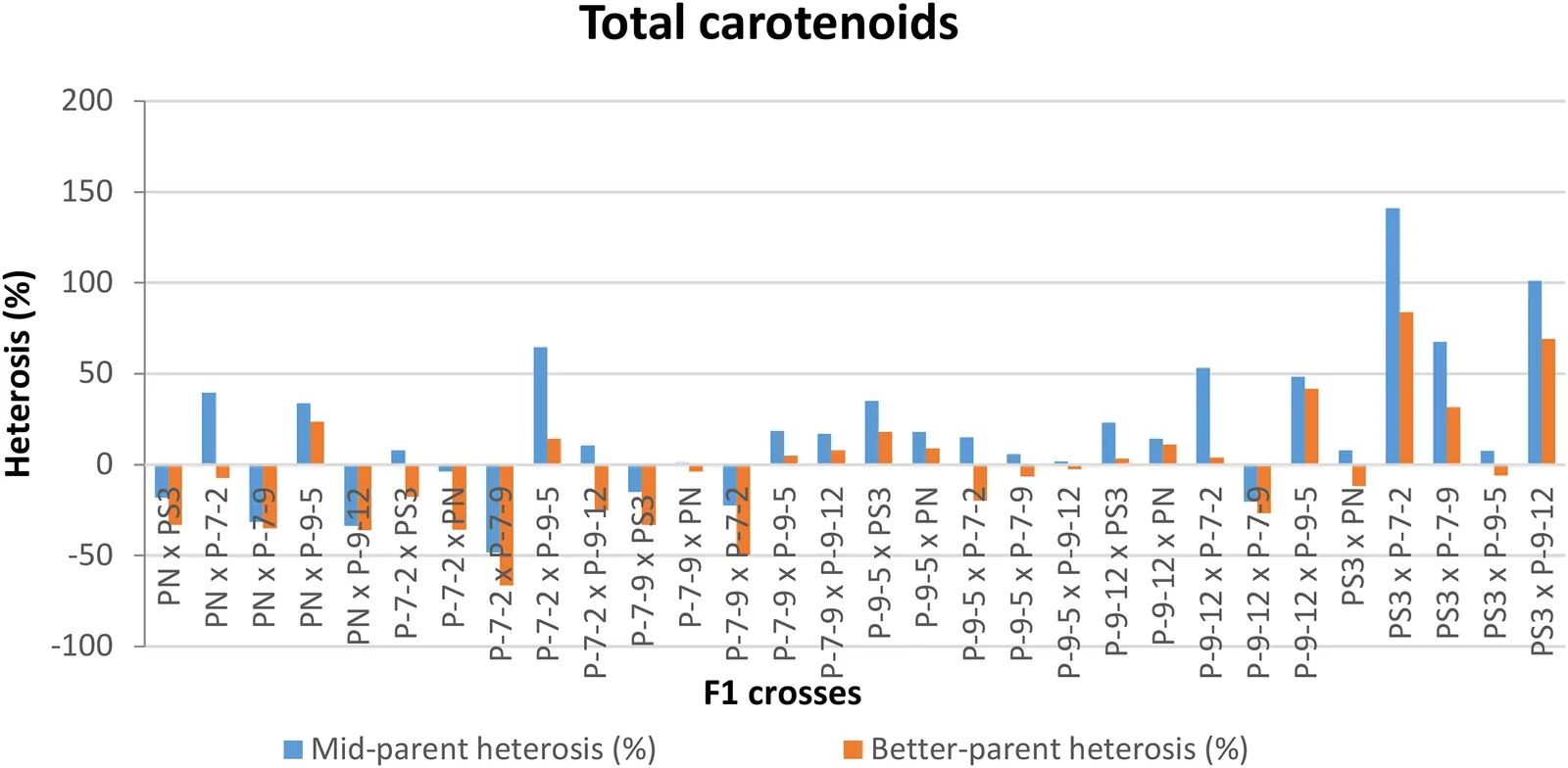
Mid-parent heterosis and better parent heterosis values in 30 F_1_ hybrids of papaya for total carotenoids.

**Figure 10:**
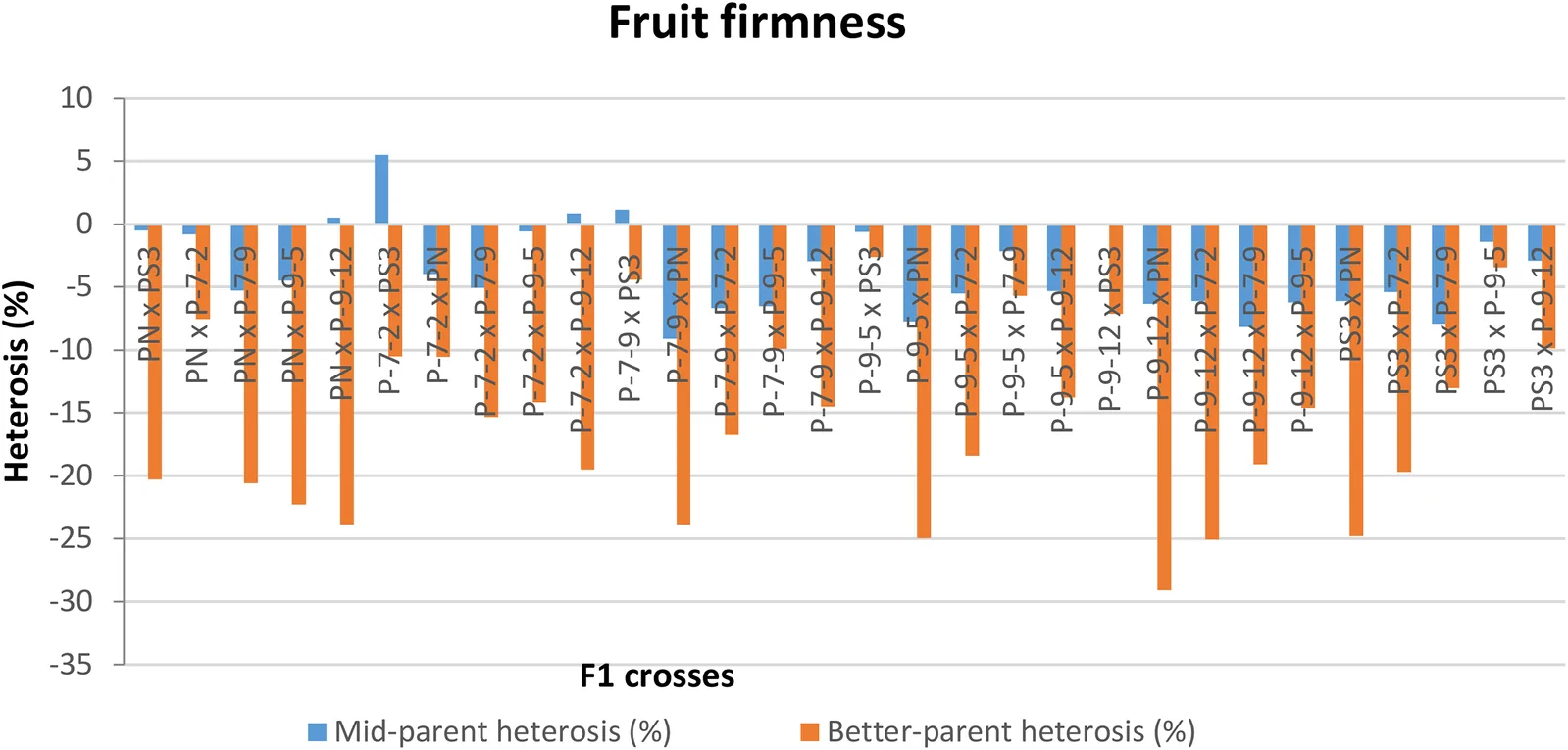
Mid-parent heterosis and better parent heterosis values in 30 F_1_ hybrids of papaya for fruit firmness.

**Figure 11:**
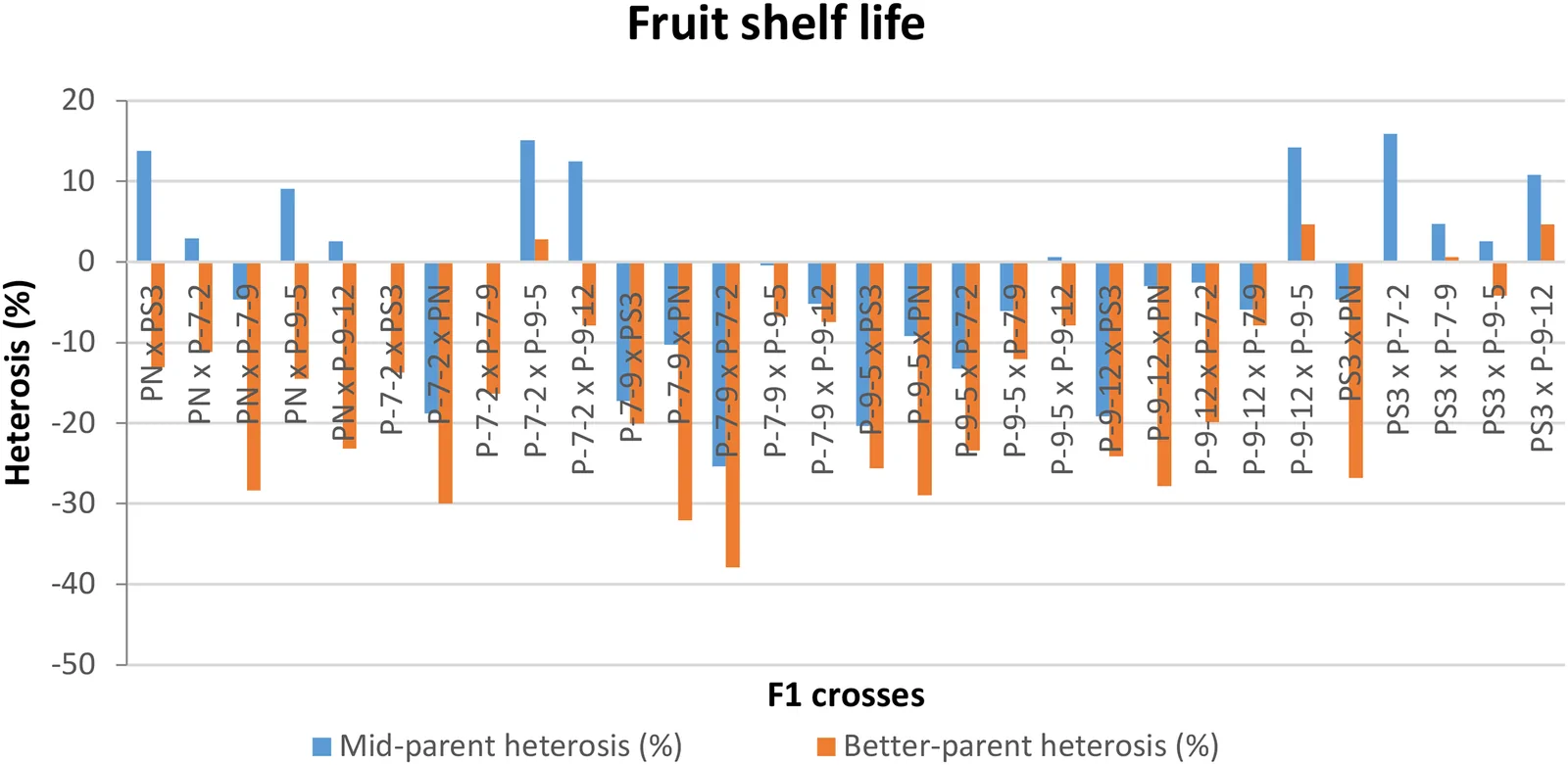
Mid-parent heterosis and better parent heterosis values in 30 F_1_ hybrids of papaya for fruit shelf life at ambient conditions.

## 4 Discussion

Papaya breeding programs worldwide have predominantly focused on improving fruit yield, with comparatively less emphasis on the enhancement of fruit quality traits. However, breeding efforts aimed at developing nutritionally superior papaya varieties must also take into account important consumer preferences such as sweetness, crispiness or mealiness, and flavor. Heterosis breeding offers a valuable approach to enhancing productivity, earliness, uniformity, quality, and resistance to pests and diseases (Riggs, 1988). This technique has led to significant improvements in various field crops, including maize, sorghum, barley, rice, and wheat, by harnessing heterosis. Beyond cereal crops, heterosis breeding has also driven notable advancements in horticultural crops such as tomato, eggplant, melons, okra, cabbage, cauliflower, and papaya. Among the fruit crops, papaya is one crop in which the phenomenon of heterosis has been exploited to some extent. In the present investigation, significant differences in fruit quality and nutritional traits revealed substantial genetic variability within the germplasm. Similar levels of variability within papaya heterotic groups have also been reported by Cardoso et al. (2014) and Vivas et al. (2018). Such genetic diversity among parental lines increases the likelihood of obtaining superior cross combinations and facilitates the effective exploitation of heterosis (Mendes et al., 2026). Furthermore, for most of the traits studied, the mean performance of hybrids exceeded that of their respective parents, indicating considerable heterosis. The PCA further supported the presence of substantial multivariate diversity, with the first three principal components accounting for 79.6% of the total variation. The fruit quality variation in the studied papaya genotypes was multidimensional, with overall fruit and nutritional quality attributes contributing predominantly to PC1, while phenolic, flavonoid and carotenoid traits were represented by PC2 and PC3, respectively. Thus, the PCA provided an integrated view of the complex relationships among fruit quality and nutritional traits and complemented the combining ability and heterosis analysis.

Total soluble solids (TSS) is an important determinant of papaya eating quality that critically influences consumer acceptance. Improvement of TSS has been the focal point of several heterosis breeding studies aimed at improving fruit quality, not only in papaya but also in crops such as strawberry (Kaczmarska et al., 2017), tomato (Liu et al., 2021; Pavan et al., 2022; Mendes et al., 2026), muskmelon (Badami et al., 2020; Kaur et al., 2022; Martins et al., 2025) and pumpkin (Hosen et al., 2022). In the present study, most cross combinations exhibited negative SCA estimates and a narrow range of variation in TSS, suggesting limited scope for non-additive gene action for this trait. Similar observations have also been reported by Ide et al. (2009); Barros et al. (2017); Santa-Catarina et al. (2019), and Miranda et al. (2025) in different papaya hybrids. Among the parental lines, P-9–12 emerged as the best parent and general combiner for TSS, indicating the importance of additive gene effects in the inheritance of this trait. The high GCA effect observed for P-9–12 suggests its suitability for utilization in selection and population improvement programs aimed at enhancing fruit sweetness. Cross combinations such as PN x P-7-2, PN x PS3, and PS3 x P-9–5 were identified as superior hybrids, exhibiting significant positive SCA effects along with desirable heterosis for TSS. The positive SCA effects observed in these hybrids indicate the involvement of favorable non-additive interactions in specific cross combinations. The simultaneous occurrence of superior GCA in parental lines and significant SCA effects in certain hybrids suggests that both additive and non-additive gene actions contribute to the inheritance of TSS in papaya. The identified parents and hybrids represent valuable genetic resources for breeding of papaya varieties with improved sweetness, enhanced fruit quality and greater consumer acceptability.

Papaya fruits are well recognized for their high antioxidant potential, primarily attributed to the presence of phenols, flavonoids, carotenoids, and lycopene. Phenols and flavonoids are important secondary plant metabolites that contribute to oxidative stability and microbial resistance and possess antioxidant, anti-carcinogenic, and anti-mutagenic properties beneficial to human health (Ribarova et al., 2005). In the present study, parental lines P-9–5 and P-7–9 recorded the highest GCA values for total phenolic content (TPC), total flavonoid content (TFC), and antioxidant capacity and also emerged as promising general combiners for these traits, indicating the predominance of favorable additive gene effects. The high GCA effects observed in these parents suggest their potential utility in selection-based breeding programs aimed at improving antioxidant-related quality traits in papaya. Interestingly, P-9–12 emerged as an excellent general combiner for TPC and antioxidant activity but exhibited a negative GCA effect for TFC. This suggests that the genetic determinants governing phenolic accumulation, flavonoid biosynthesis, and overall antioxidant capacity may differ, highlighting the importance of selecting complementary parents to simultaneously improve multiple nutraceutical traits. Antioxidant activity was particularly high in hybrids such as P-9–5 x P-9-12, P-9–12 x P-9-5, P-9–5 x P-7-9, and P-9–12 x P-7-9, suggesting the successful accumulation of favorable alleles governing antioxidant-related traits. The simultaneous occurrence of high GCA in parents and significant SCA effects in specific hybrids suggests the involvement of both additive and non-additive genetic components in the inheritance of antioxidant traits. The PCA results complemented these findings, as phenolic and flavonoid traits showed strong association with PC2. The positioning of genotypes along PC2 therefore reflected differences in their phenolic-flavonoid profiles. In particular, P-7–9 x P-9-5, which recorded the highest TPC and TFC values, represented a promising combination for enhancing nutraceutical quality. Collectively, these findings indicate that selection of complementary parents is important for simultaneous improvement of related traits.

The fruits of papaya are also a rich source of provitamin A carotenoids, with red pulp varieties particularly high in lycopene along with β-carotene and β-cryptoxanthin, whereas the yellow pulp varieties are devoid of lycopene (Schweiggert et al., 2012). Parental lines P-7–9 and P-9–12 contained high levels of β-carotene, lycopene, and total carotenoids, and P-9-12, an orange-pink-colored line, was the best general combiner for all three pigment-related traits. Hybrids such as P-9–12 x P-7-9, P-7–9 x P-9-12, and P-9–12 x P-9–5 showed elevated levels of β-carotene, lycopene, and total carotenoids, thereby indicating the potential to develop nutritionally enriched hybrids with enhanced antioxidant properties. Similarly, a study of Costa Rican papaya for carotenoid profiling revealed significant genetic variability among the lines and hybrids (Schweiggert et al., 2012). Similar studies for estimation of combining ability and heterosis for carotenoids have been carried out in cauliflower (Dey et al., 2014), pumpkin (Hosen et al., 2022), muskmelon (Kaur et al., 2022) and Tunisian melon (Chikh-Rouhou et al., 2024). The moderate level of heterosis and low SCA effects observed for lycopene, β-carotene and total carotenoids in papaya suggest a greater influence of additive genetic effects in their inheritance. Variation in heterosis for lycopene and β-carotene may also result from complementary interactions among parental alleles influencing carotenoid biosynthesis and metabolic activity in hybrids. Carotenoid accumulation is regulated through coordinated enzymatic steps affecting precursor availability and pathway flux and may also be influenced by environmental conditions. The PCA further indicated that lycopene, β-carotene and total carotenoids contributed strongly to PC1 and PC 3, with PC3 showing particularly strong associations with lycopene and total carotenoids. The relatively high PC3 score of PS3 x P-9–12 indicates its distinct carotenoid-related profile, while the favorable PC scores of several P-9-12-derived hybrids were consistent with their superior carotenoid concentrations. Thus, PCA provided additional evidence for the importance of these crosses as potential sources of nutritionally valuable carotenoid combinations.

Fruit firmness, an important trait positively associated with shelf life, handling, and transportability of fruits (Singh et al., 2019), exhibited negative SCA effects, along with negative mid-parent heterosis and heterobeltiosis, in the majority of hybrid combinations evaluated in the present study. This suggests a limited contribution of non-additive gene action towards the improvement of firmness and indicates the predominance of additive genetic effects for these traits. Analysis of GCA effects identified only two parental lines, namely P-9–5 and P-9-12, as positive general combiners for fruit firmness and shelf life, highlighting their potential utility in breeding programs aimed at improving post-harvest quality. Among the hybrids, combinations such as P-9–12 x PS3, PS3 x P-9-12, P-9–5 x P-9-12, P-7–9 x P-9-12, and P-9–12 x P-9–5 exhibited comparatively superior fruit firmness and extended shelf life. The superior performance of these hybrids may be attributed to the favorable accumulation of additive gene effects contributed by the parental lines. The strong positive contribution of firmness and shelf life to PC1 further demonstrated that postharvest performance constituted an important component of overall multivariate fruit quality. The high PC1 score of P-9–12 x P-9–5 is consistent with its favorable combination of several quality and postharvest attributes, supporting its potential as a promising hybrid for further evaluation. Similar observations on superior firmness in specific hybrid combinations have also been reported by Miranda et al. (2025) in papaya, where combinations SS-72/12 x UCLA08–060 and SS-72/12 x UCLA08–096 from the intermediate group and SS-72/12 x UCLA08-026, SS-72/12 x UCLA08–013 and SS-72/12 x UCLA08–097 from the Formosa group exhibited high fruit firmness. Improved fruit firmness is particularly desirable in papaya, as it enhances resistance to mechanical damage during handling and transportation, thereby extending marketability and reducing post-harvest losses. Considering the greater role of additive gene effects governing these traits, effective selection for desirable fruit texture, firmness, and shelf-life can be achieved in this population.

The present study demonstrates that combining ability and heterosis analyses provide an effective framework for identifying superior parental lines and hybrid combinations for simultaneous improvement of nutritional and postharvest quality traits in papaya. The observed variation in combining ability among the parental lines and the trait-specific superiority of different hybrids indicate that both additive and non-additive genetic effects contribute to fruit quality improvement, although their relative importance varies among traits. These findings also emphasize that no single parent or hybrid was superior for all evaluated attributes, highlighting the need for strategic parent selection based on specific breeding objectives. More broadly, the integration of combining ability, heterosis and PCA provides a transferable framework for fruit breeding programs targeting complex quality traits, functional foods and adaptation to changing production environments. As the present investigation was conducted using a limited set of selected elite inbred lines under a single growing season and location, further validation across diverse environments and broader germplasm is required to confirm the stability of the identified combinations. Integration of molecular approaches with conventional breeding will further facilitate the development of nutritionally superior papaya hybrids with improved postharvest performance.

## 5 Conclusion

The present investigation demonstrated substantial genetic variability among the evaluated papaya inbred lines and their hybrids for fruit nutritional quality and shelf-life traits, confirming the potential of combining ability analysis and heterosis as an effective approach for identifying superior parents and hybrid combinations. P-9–12 emerged as the most promising general combiner for total soluble solids, antioxidant activity, carotenoid pigments, fruit firmness, and shelf life, while P-9–5 and P-7–9 were identified as valuable donors for improving phenolic and flavonoid contents. The study also revealed that additive gene action predominated for several quality and postharvest traits, whereas non-additive gene action played a significant role in the expression of antioxidant-related attributes, as reflected by the superior performance of specific hybrid combinations. Hybrids such as P-9–12 x PS3, P-9–12 x P-9-5, P-9–5 x P-9-12, P-7–9 x P-9-5, PN x PS3, and PN x P-7–2 were identified as promising candidates for improving fruit quality, nutritional composition, and shelf life. Principal component analysis further confirmed the multidimensional nature of fruit quality variation and effectively differentiated parents and hybrids based on their overall nutritional and postharvest attributes. These findings provide valuable genetic resources and practical guidance for developing high-quality papaya hybrids and formulating future breeding strategies aimed at enhancing fruit nutritional value, postharvest performance and resilience to changing production environments.

## Data Availability

The original contributions presented in the study are included in the article/supplementary material. Further inquiries can be directed to the corresponding author.
